# Influencing Factors of Environmental Risk Perception during the COVID-19 Epidemic in China

**DOI:** 10.3390/ijerph18179375

**Published:** 2021-09-05

**Authors:** Jingfei Zhang, Zhicheng Zheng, Lijun Zhang, Yaochen Qin, Jieran Duan, Anyi Zhang

**Affiliations:** 1 College of Geography and Environmental Science, Henan University, Kaifeng 475004, China; 18211719372@163.com (J.Z.); zhengzhicheng@henu.edu.cn (Z.Z.); zlj7happy@vip.henu.edu.cn (L.Z.); duanjieran0419@163.com (J.D.); 104753190118@henu.edu.cn (A.Z.); 2 Key Laboratory of Geospatial Technology for the Middle and Lower Yellow River Regions, Henan University, Kaifeng 475004, China; 3 Key Research Institute of Yellow River Civilization and Sustainable Development and Collaborative Innovation Center on Yellow River Civilization Jointly Built by Henan Province and Ministry of Education, Henan University, Kaifeng 475004, China

**Keywords:** COVID-19, environmental risk perception, spatial-temporal big data, resilient city, order logistic regression, China

## Abstract

The spread of COVID-19 is having a serious impact on socioeconomic development, and increased environmental risk perception (ERP). ERP provide new ideas for the orderly recovery of society. However, there have been studies that often pay attention to individual factors, and less concerned about the external environment. In fact, ERP will be affected by the external environment and individual factors. We used a Python script to collect 65,277 valid Weibo comments during the COVID-19 epidemic in China to assess urban residents’ environmental risk perception (ERP). SnowNLP emotion analysis was used to measure the ERP of 366 urban in China, and the structural proportion characteristics and spatial-temporal differentiation of ERP were analyzed. Then, an order logistic regression model was used to investigate the relationship between economic level, social security, medical facilities and ERP. The study investigated the Chinese cities have a higher ERP during the COVID-19 period, and it shows marked fluctuations. As COVID-19 spreads, the ERP shows a distribution pattern of “high in the southeast and low in the northwest” with Hu line as the boundary and “from high to low” with Wuhan as the high value center. COVID-19 serves as catalysts for ERP, the impact of COVID-19 is enhanced after socioeconomic factors are considered. The economic level effectively regulates ERP, except the stage of accelerating diffusion. ERP is effectively stabilized by social security and medical facilities. After considering all the variables simultaneously, we found that the mitigation effect of social security and medical facilities on ERP has improved.

## 1. Introduction

COVID-19 is widely regarded as the world’s biggest crisis since the Second World War [1,2]. The World Health Organization (WHO) announced that COVID-19 had reached pandemic proportions on 11 March 2020 [1,3]. This Public Health Emergency of International Concern (PHEIC) has a negative impact on not only social and economic development but also individuals’ physical and mental health [4,5], causing fear, depression and panic [6,7,8]. In addition, many countries and regions have been forced to take measures such as city closures, business suspensions, school suspensions and other steps to limit people’s movement and maintain social distance and isolation in order to combat the spread of COVID-19 [9]. This will change the living habits and consumption behaviors of residents and affect their emotional state indirectly [10,11,12]. Some scholars have confirmed that crisis events such as economic crises, natural disasters, conflicts and terrorist attacks can cause ERP [13]. COVID-19, similar to the crises above-mentioned, causes serious economic losses and casualties, and affects the public’s ERP [3,13]. The public’s anxiety, fear and depression during COVID-19 are explicit manifestations of ERP [13,14,15].

ERP is a kind of risk perception, is the public’s subjective judgment on the characteristics and severity of objective risks in the surrounding environment [16,17]. ERP is a cognitive structure that can’t exist independently of the external environment [13,18,19,20]. ERP is influenced by the nature of risk, subjective attitude, individual attributes, economic status and social background [16], and the interaction of psychological processes, social environment and institutional factors can change people’s understanding of risk [21]. The cultural theory used to explain ERP also emphasizes the importance of society and community [21]. The city is a collection of population, materials, economic and social interactions, and its socioeconomic factors can influence not only how environmental risks are perceived but also how they are ameliorated [22]. We can adopt a scientific and rational approach to deal with the crisis by investigating the residents’ ERP in different regions in the face of crisis events [11]. Therefore, the influencing factors of urban socioeconomic attributes on ERP have become the focus of geography, sociology and psychology.

Previous studies have focused on ERP under COVID-19 and its influencing factors. Relevant scholars mainly pay attention to the ERP of specific groups such as pregnant women, staff, hospitalized patients and medical staff and so on [8,23,24,25,26]. Most of these studies have consisted of qualitative research and quantitative research. The relevant researchers used the risk perception scale to evaluate the ERP during COVID-19 [13,23,27], and use multiple linear regression and structural equation model to explore its influencing factors [13,25]. The influencing factors of ERP are mainly divided into the following three categories: (1) Psychological factors: loneliness, attitude and knowledge significantly affect the ERP of residents during COVID-19 [28]. (2) Socioeconomic factors: Gender, education level, marital status, occupation, monthly income and age affects residents’ views on COVID-19 [25,28]. (3) Policies and measures: Social isolation and public health measures indirectly affect resident’s ERP [12].

After reviewing the prior literature, we found that the relevant research on ERP during COVID-19 has the following shortcomings. (1) Most of the existing studies explored the influencing factors of ERP during the COVID-19 pandemic from the perspectives of psychology, sociology and medicine, while few studies have analyzed its spatial-temporal pattern and influencing factors from the perspective of geography. (2) Most of the relevant studies have been based on traditional survey data, and big data have not been used. Between these, survey data is more time-consuming and laborious, while big data is more convenient and efficient, and acquires more ways to obtain data. (3) Most of the existing studies focus on individual ERP, less on urban ERP. (4) Existing studies have mainly focused on psychological factors and demographic characteristics while neglecting urban socioeconomic environment and other external factors.

Based on these considerations, Python was used to crawl the text and user locations of Weibo comments under postings from CCTV News and People’s Daily during China’s COVID-19 outbreak (8 December 2019–8 April 2020). Then the SnowNLP emotional analysis tool was used to calculate the ERP of each city. Finally, the confirmed and death of COVID-19, economic level, social security and medical facilities were incorporated into an ordered logistics model to explore the direct and interactive effects of each variable on the ERP. The research questions of this paper are as follows: (1) What are the spatial-temporal and structural characteristics of the ERP of Chinese cities under COVID-19? (2) Will the urban socioeconomic factors, confirmed and deaths of COVID-19 affect the ERP of each city? (3) Does the urban socioeconomic factors interact with spread of COVID-19 in affecting ERPs? If so, what is the degree of interaction? This study makes two key contributions. First, spatial-temporal big data mining technology was used to measure the ERPs of cities. Secondly, economic level, social security and medical facilities were incorporated into the model to explore the socioeconomic drivers of ERP.

The rest of the paper is organized as follows: Section 2 introduces the research design, and research hypothesis. Section 3 describes the data source, variable selection and research methods. Section 4 introduces the results of our study. Section 5 presents a discussion of the results. Finally, the key conclusions of this paper and corresponding policy recommendations are presented.

## 2. Research Design and Model Method

### 2.1. Research Design

Geographers believe that perception is how people feel about the external environment of specific things, and it emphasizes the immediate understanding of environmental information [29]. Environmental perception is the individual’s consciousness or perception of their surroundings, that is, the psychological process in which the daily living environment forms an overall impression in the brain [30,31]. The individual’s psychological perception and cognitive structure are influenced by the external environment (social and physical environment) [32]. From the perspective, environmental psychology focuses on external environmental (social environment and physical environment) on individual psychology [33]. From theory, behavioral background theory believes that external environments affect individual life attitude [34]. Distributed cognitive theory will turn to life context and analyze the impact of external environment and social culture on cognitive activities [18]. ERP is an environmentally awareness that is affected by external environments and individual factors [18,19,20], and the city is the place of residents’ everyday lives, and its economic level, social security, medical facilities and other external environment will regulate residents’ ERP.

Infrastructure, institutional, economic and social are the components of a city [35], and they cover the economic, social, institutional, environmental and other aspects of the city [36]. The economic level is the foundation and driving force of urban. Social security is the guarantee of urban sustainable development. Infrastructure is a special urban artificial environment, which is the key factor to ensure the resilience of human and urban environmental systems [36,37]. COVID-19 is considered as a starting point for rethinking and reshaping the urban environment, as well as an opportunity for cities to carry out resilience, inclusiveness and ecological transformation [10,38]. The outbreak of the COVID-19 epidemic will reflect expose the fragility vulnerability and resilience of urban structures [39]. The ERP provides a new thinking point for resilient urban construction. Therefore, we focus on the effects of urban economic level, social security and medical facilities on ERP, which allows us to investigate the city’s shortcomings and propose new policy recommendations for the planning and development of resilient cities.

ERP is not only the residents’ response to COVID-19, but also influenced by urban socioeconomic variables such as economic level, social security and medical facilities, which have the characteristics of variability and sociality. In order to reflect this variability, the COVID-19 epidemic was divided into four stages: early dissemination, accelerating diffusion, decelerating diffusion and prevention and control. In order to reflect sociality, variables relating to economic level, social security and medical facilities were put into the ordered logistic model to analyze the influencing factors of ERP. It should be noted that we divided the COVID-19 epidemic into the following four phases: (1) Early dissemination stage: 8 December 2019–20 January 2020. (2) Accelerating diffusion stage: 20 January 2020–23 February 2020. (3) Decelerating diffusion stage: 23 February 2020–20 March 2020. (4) Prevention and control stage: 20 March 2020–8 April 2020 [40].

**Figure 1:**
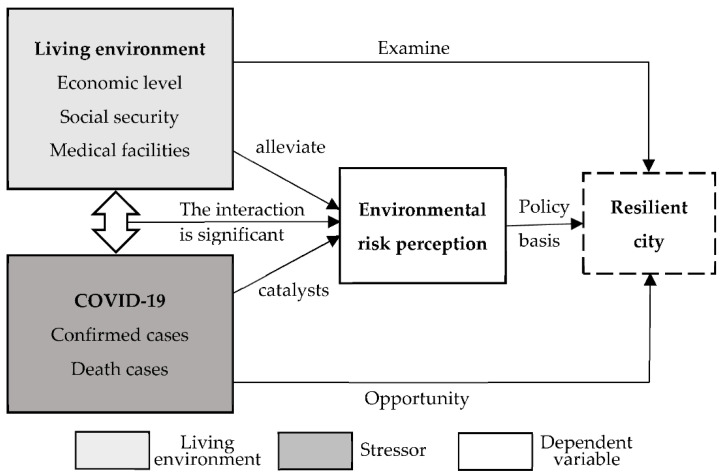
Analysis framework of environmental risk perception during COVID-19.

COVID-19 is a stressor, as shown by the above. Residents are concerned about the confirmation and death of COVID-19, as well as the measures taken by the relevant departments to control the spread of COVID-19, such as homework and social distance. Residents’ panic and anxiety may be intensified as a result of these events [12]. Therefore, we consider COVID-19 as the stressor and the variables such as economic level, social security and medical facilities as a living environment to construct an analysis framework (Figure 1) to investigate the factors influencing ERP during COVID-19.

### 2.2. Research Hypothesis

Figure 1 shows how ERP serves as a policy foundation for the construction of resilient cities in the post-epidemic era. Figure 1 shows how ERP serves as a policy foundation for the construction of resilient cities in the post-epidemic era. In terms of the relationship among living environment, COVID-19 and ERP, urban economic level, social security and medical facilities represent urban socioeconomic factors, which can effectively alleviate ERP during COVID-19. The confirmed and death of COVID-19 are the catalysts of ERP, which will further aggravate the public’s ERP. COVID-19 is an opportunity for the development of resilient cities, while economic level, social security and medical facilities provide policy foundations for resilient cities.

The spread of COVID-19 is a direct threat to public health and safety, and the panic and insecurity generated by deaths are even more devastating. Based on this, Hypotheses 1 (H1) and (H1a) are proposed.

**Hypothesis** **1** **(H1).** *The spread of COVID-19 will aggravate residents’ ERP.*

**Hypothesis** **1** **(H1a).** *The effect of deaths cases on ERP under COVID-19 is more obvious than that of confirmed COVID-19 cases, and the degree of influence is also greater.*

COVID-19 is a social as well as a medical event. As a result of the spread of COVID-19, social changes such as economic recession and increased unemployment would exacerbate ERP. Cities with a better socioeconomic environment have better resilience and flexibility, which will ultimately alleviate residents’ ERP. Residents living in cities with a better resilience are expected to have a more stable and positive view of environmental risk during COVID-19. Therefore, Hypothesis 2 (H2) is proposed.

**Hypothesis** **2** **(H2).** *The mitigation effect of social security and medical facilities on ERP has improved.*

Interaction is the core of the analytical framework, which refers to the influencing factors of ERP after considering variables such as COVID-19, economic level, social security and medical facilities. The spread of COVID-19 emphasizes the importance of socioeconomic factors in disease prevention. Therefore, we propose Hypotheses 3 (H3), (H3a) and (H3b).

**Hypothesis** **3** **(H3).** *The faster the spread of COVID-19, the more significant the mitigation effect of economic level, social security and medical facilities on ERP.*

**Hypothesis** **3** **(H3a).** *The interaction between confirmed cases and socioeconomic variables is more obvious than the interaction between death cases and socioeconomic variables.*

**Hypothesis** **3** **(H3b).** *Among the COVID-19 interactions, social security and medical facilities play a significant role in alleviating ERP.*

## 3. Data and Methods

### 3.1. Data

#### 3.1.1. Weibo Comments Data

In China, Sina microblog is similar to Twitter. During the COVID-19 period, 6.076 million authoritative information about the epidemic situation were released through Weibo, and the number of readings by residents reached 364.7 billion. A total of 30,000 live broadcasts of the epidemic were launched, with more than 3 billion viewers. According to Sina microblog user development report in 2020, the number of monthly active users of Sina microblog in September 2020 was 511 million. In terms of age, 30% of users are under 20 years old, 66% are between 20 and 40 years old, and 4% are above 40 years old. From the perspective of gender characteristics, male users account for 45.4%, and female users account for 54.6%. Many scholars choose hot online news according to themes and the number of comments to analyze the public perception of social events [41]. Therefore, the ERP in this paper comes from Weibo comments data. In this study, we use the Weibo comments under postings from CCTV News and People’s Daily during China’s COVID-19 outbreak (8 December 2019–8 April 2020) as the research data.

**Figure 2:**
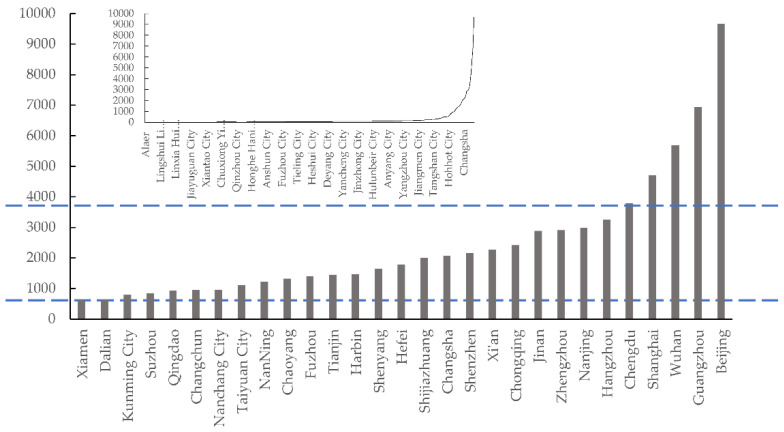
Number of urban microblogging comments.

The Weibo comments data acquisition was mainly divided into the following three steps. First, based on the research design, we divide the COVID-19 period in China into four stages. Second, select Weibo related to the number of COVID-19 in China posted by CCTV News or People’s Daily within the time frame, and crawl the text of user comments under each Weibo. Third, crawl the city information of the commenting users. It can be seen from Figure 2 that after obtaining the number of comments in each city, we can see that the number of Weibo comments in most cities is 0–100. From the num of comments, there are 22 cities more than 1000 and there are 4 cities (Shanghai, Wuhan, Guangzhou and Beijing) more than 4000.

#### 3.1.2. Statistical Data

Urban socioeconomic factors. The urban socioeconomic factors in this paper were derived from the China Statistical Yearbook for 2019. Due to the delay in statistical yearbook data, when writing this paper, the relevant data for 2019 had not yet been released. Combined with the previous year data, we found the socioeconomic factors of each city have arguably changed little in a year. In addition, our research object is ERP, and residents’ perception of urban socioeconomic attributes is a one-sided. Therefore, 2018 data were used as socioeconomic factors.

COVID-19. The COVID-19 data are derived from the National Health Commission of the People’s Republic of China, and the COVID-19 indicators of the four stages are calculated based on the data.

### 3.2. Variable Selection

#### 3.2.1. Dependent Variable

The ERP of each city is the dependent variable. The data processing of ERP includes the following five steps. First, the acquisition of data by a Python script that gains 128,529 Weibo comment data points. The second stage is data cleaning, which entails removing missing data, removing duplicates and filtering short sentences to obtain a valid text. It mainly includes the following three small steps: (1) Comments with empty text were deleted. (2) Duplicate entries were deleted. (3) Comments with less than two Chinese characters in the text were removed. The third step is to do an emotional analysis. SnowNLP, which is used in data processing, is a database for word segmentation, part-of-speech tagging and emotional analysis. It mainly determines the emotional state of individuals by analyzing and mining text content. The range of emotional state scores is between 0 and 1. Generally speaking, when the value is greater than 0.5, the emotion is more positive. When the value is less than 0.5, the emotion is more negative [42]. The fourth step is the ERP. When the emotional state is positive, it indicates that the ERP is low. The fifth step is to calculate the city ERP. The ERP obtained by SnowNLP is a single ERP for each comment. To obtain the city’s ERP, we calculate the average value based on the reviewers’ city.

The above is indicated that the use of SnowNLP measurement of emotional status scores are the basis for subsequent research. Next, we will give an example of the calculation step of the SnowNLP emotional state: First, acquire text data. ——“too terrifying”. Second, use the Jieba tool to distinguish the text content and labeled the words. ——“too (adverb), terrifying (adjective)”. Third, define the emotional dictionary and extract the emotional words of each line of text. ——“too (80%), terrifying (0.3)”. Fourth, build an emotional matrix using emotions and calculate emotional scores. ——“0.3 * 80% = 0.24”.

**Figure 3:**
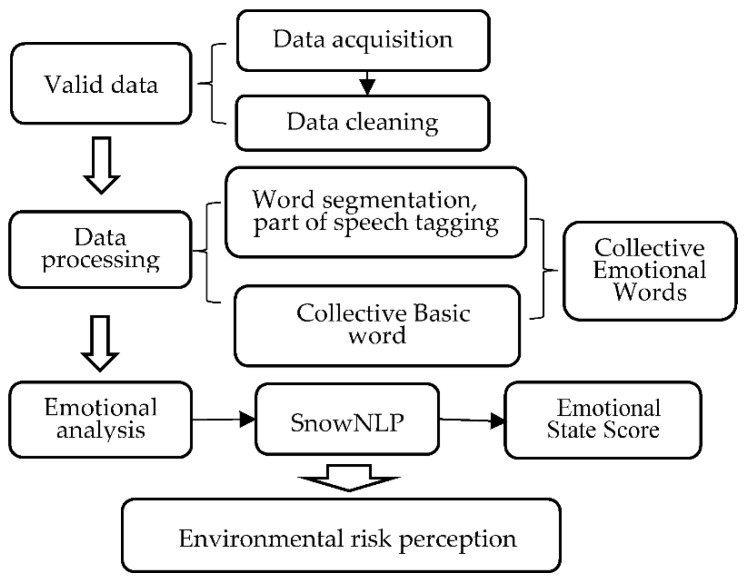
Data processing of environmental risk perception.

We reclassified the values of emotional status above 0.5 according to passing and excellent scores in order to further refine the ERP [43]. ERP is divided into the following four categories. First, the emotional state score is from 0 to 0.5, which is the highest. Second, the emotional state score is 0.5 to 0.6, which is higher. Third, the emotional state score is 0.6 to 0.8, which is low. Fourth, the emotional state score is 0.8 to 1, which is the lowest. The flow chart of data processing is shown in Figure 3:

#### 3.2.2. Independent Variable

Based on the above, 14 variables representing economic level, social security, medical facilities and COVID-19 were selected (Table 1).

(1) COVID-19 variables

The independent variables in this study are cumulative confirmed cases, growth rate of confirmed cases, cumulative death cases and death rate. There was often a value of 0 for confirmed COVID-19 cases and deaths in each city based on the daily epidemic data published by the National Health Commission of the People’s Republic of China. In the period of the spread of COVID-19, residents are highly concerned and nervous, and the COVID-19 situation of the province will significantly affect the ERP of individuals living in that province. In order to avoid the occurrence of zero value and improve the effectiveness of the model results. Therefore, we take the COVID-19 data of each province as the basic COVID-19 data of each city to carry on the research. The main processing steps are divided into the following steps. First calculate the COVID-19 diagnosis and death of each province. Then classify the city according to the provinces of each city. Finally, the COVID-19 variable value of each city is the COVID-19 variable value of the province. Among Table 1, the confirmed and death variables are different during COVID-19.

(2) Urban socioeconomic factors

ERP is essentially a kind of environmental perception, and it emphasizes the feel about the external environmental information. The economic level, social security and medical facilities of city affect the ERP during COVID-19. So, we explore the influencing factors of ERP during COVID-19 from three aspects: economic level, social security and medical facilities. Among these, the economic level includes resident deposit balance and average wage. Social security includes population density, endowment insurance, medical insurance, unemployment insurance and unemployment registration. Medical facilities include hospitals, hospital beds and doctors. Table 1 presents the qualitative description of each indicator.

### 3.3. Methods

#### 3.3.1. Exploratory Spatial Data Analysis

The first law of geography considers that adjacent areas are more similar in geographic phenomena [43]. Among them, the ESDA is mainly to perform visualization processing, indicating the similarity of the attribute value of the space proximity area. Therefore, we use GEODA to analyze the spatial agglomeration characteristics of ERP in each city. The relevant calculation formula is as follows:

(1) $$I=\frac{\sum_{i=1}^{n}\sum_{j=1}^{n}W_{\mathrm{ij}}{(X}_{i}{-\;\bar{X})(X}_{j}-\bar{X})}{S^{2}\sum_{i=1}^{n}\sum_{j=1}^{n}W_{\mathrm{ij}}}$$

(2) $$S=\frac{\sum_{i=1}^{n}{{(X}_{i}-\bar{X})}^{2}}{n}$$

In Formulas (1) and (2), *I* is Moran’I,${\;W}_{\mathrm{ij}}$ is the spatial weight of city *i* and *j*. $X_{i}$ and $X_{j}$ are ERP of city *i* and *j*. $\bar{X}$ is a mean of ERP, *I*, *j* = 1, 2, ……, 366. The value of the Moran’I is −1 to 1. When creating space weight, we select Queen’s space neighboring method.

Moran’I ignores the potential instability problem of spatial processes when exploring spatial heterogeneity. The local self-relevant can effectively analyze the high-value gathering area of ERP, it is impossible to make up for this shortage. Among them, the calculation formula is as follows:

(3) $$I_{i}=\frac{\left(X_{i}-\bar{X}\right)}{S^{2}}\sum_{i\neq j,j=1}^{n}W_{\mathrm{ij}}{(X}_{i}-\bar{X})$$

#### 3.3.2. Order Logistic Regression Model

According to the measurement results, the ERP was ranked as 1 = lowest ERP, 2 = lower ERP, 3 = higher ERP, 4 = highest ERP. Since the dependent variable “ERP” is a four-classified ordered variable, an ordered logistics model was used to explore the influencing factors of ERP. The ordered logistics model is as follows:

(4) $$P_{j}=P\left(y\ll j|X\right)=\frac{\exp(a_{j}+\sum_{i=1}^{n}\beta_{i}X_{i})}{1+\exp(a_{j}+\sum_{i=1}^{n}\beta_{i}X_{i})}$$

In Formula (4), *y* represents the ERP of each city; $a_{j}$ is a constant regression coefficient;$\;\beta_{i}$ is a coefficient; $X_{i}$ represents an independent variable; *j* = 1, 2, 3, 4 represents ERP [44].

(5) $$log\frac{P_{j}}{1-P_{j}}=a_{j}+\sum_{i=1}^{n}\beta_{i}x_{i}$$

In Formula (5), *P*/1 − *P* is used to express the occurrence ratio, that is, the ratio of the occurrence probability of an event (y≪j) to the probability of non-occurrence of the event (y>j). The occurrence ratio shows the influence of the independent variable on the change in the probability of the dependent variable [44].

## 4. Results Analysis

### 4.1. Characteristic Analysis

#### 4.1.1. Structural Features

**Figure 4:**
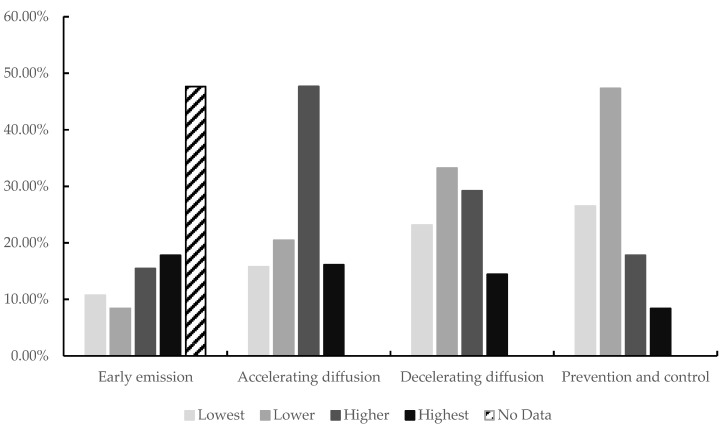
Proportion of the environmental risk perception during the COVID-19 epidemic.

The ERP of each city fluctuated significantly during the COVID-19 epidemic, particularly in the stages of accelerating diffusion and decelerating diffusion. As we can see from Figure 4, the number of confirmed and death of COVID-19 increased rapidly during the accelerating diffusion phase, with the number of cities having a highest and higher ERP exceeding 60%. In the decelerating diffusion phase, the ERP of the residents was found to have eased as COVID-19 was brought under control, and the proportion distribution tends to be uniform. The number of cities in the highest or higher ERP was 43%, and the number of cities in the lower ERP was 33.2%. In the prevention and control stage, In the prevention and control stage, COVID-19 was brought under control, and the proportion of lowest, lower, higher and highest ERP of urban is 26.51%, 47.32%, 17.79% and 8.39%, respectively.

#### 4.1.2. Spatial-Temporal Structure Characteristics

**Figure 5:**
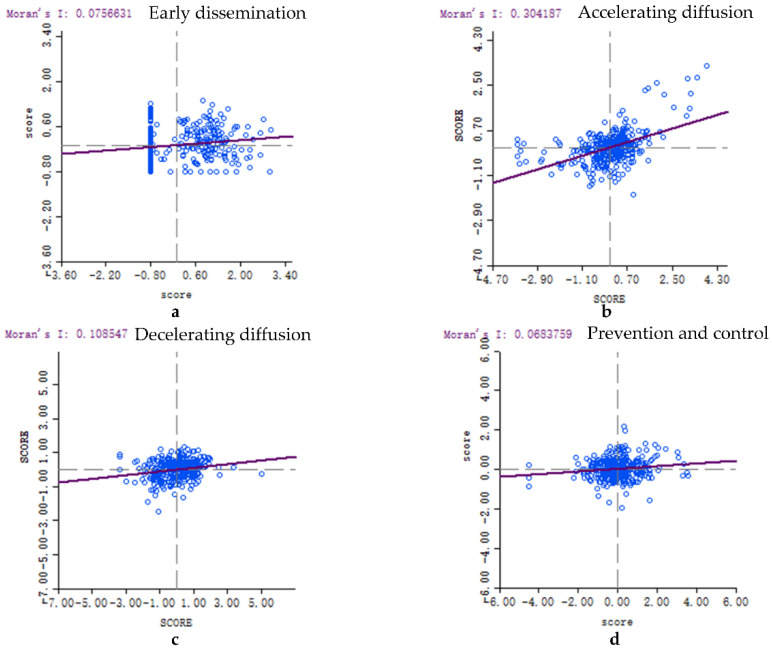
Moran scatter plot of environmental risk perception during COVID-19.

We first use exploratory space analysis to analyze the spatial autocorrelation of ERP. As can be seen from Figure 5, the Moran’I is greater than 0 during COVID-19, indicating that the ERP of adjacent cities tends to spatially agglomeration. Among them, the spatial aggregation phenomenon of accelerating diffusion during COVID-19 is more obvious.

**Figure 6:**
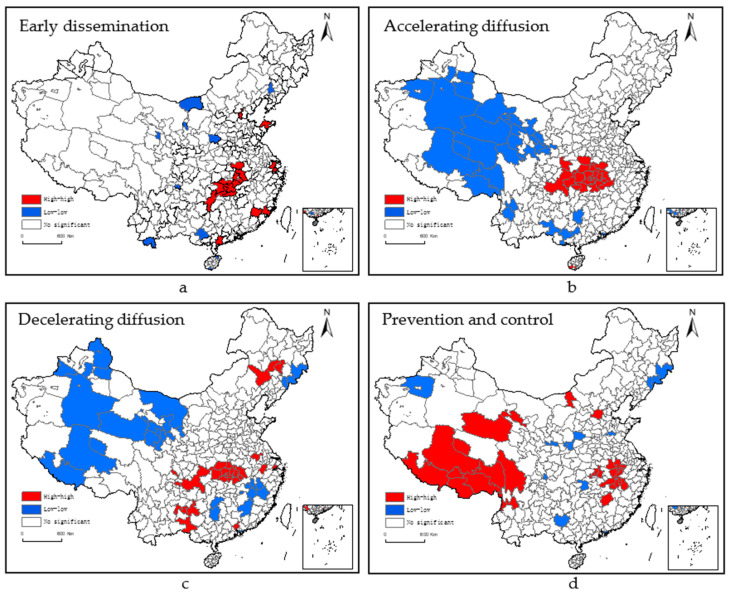
Local indicators of spatial association (Lisa)diagram of local spatial concentration of environmental risk perception.

We can be seen from Figure 5, and ERP in each city has local spatial instability. Therefore, we combine it with the LISA significance to further explore local space autocorrelation between different regions. As can be seen from Figure 6, ERP presents significantly high-high value aggregation and low-low value aggregation during COVID-19 period. Among them, the Hubei area centered on Wuhan is a clear high-value gathering area, and the western region such as Xinjiang, Tibet is a low-value gathering area. Among them, Wuhan-centered Hubei area is a clear high-high value gathering area, and the western region such as Xinjiang, Tibet is a low-low value aggregation area.

#### 4.1.3. Spatial-Temporal Distribution Characteristics

We are known from the results of Section 4.1.2, and the ERP of each city has a significant spatial autocorrelation during COVID-19. According to this result, we use the inverse distance weight (IDW) in ArcGIS 10.3 to interpolate ERP, further analyze its spatial-temporal distribution characteristics.

**Figure 7:**
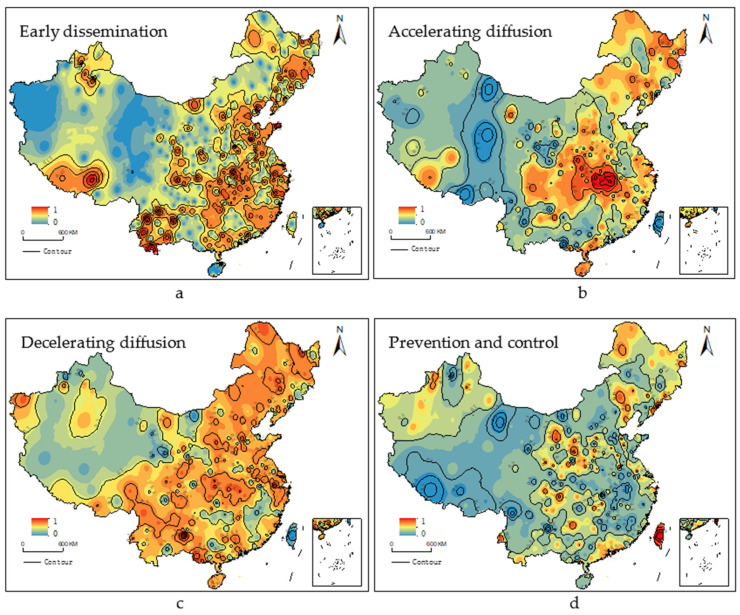
Spatial distribution of environmental risk perception during COVID-19 epidemic.

Among them, a,b,c,d in Figure 7 represent the spatial-temporal distribution of the ERPs in the early dissemination, accelerating diffusion, decelerating diffusion and prevention and control stages, respectively. The spatial distribution of ERP is different during the COVID-19 epidemic. In addition, in the period of COVID-19 diffusion (accelerating diffusion and decelerating diffusion stage), the spatial distribution pattern was found to have two characteristics. First, the ERP of China shows a distribution that was high in the east and low in the west across the Hu Line (Figure 7b,c). Second, the ERP of China shows a circular distribution pattern from high to low with Wuhan as the high value center (Figure 7b,c). However, after COVID-19 was effectively brought under control, the ERP of China improved, but the boundary of spatial distribution was not clear (Figure 7d).

From Figure 7a, only some cities pay attention to the COVID-19, although the spatial distribution of ERPs in the early dissemination stage of COVID-19 is not clear, there is an obvious high value area, that is Hubei area with Wuhan as the center has higher ERP. As shown in Figure 7b, the spatial distribution of ERPs in the accelerating diffusion stage of COVID-19 shows the following two characteristics. First, the ERP of various cities in China shows a distribution pattern of high in the southeast and low in the northwest across the Hu line. The Hu line, also known as the Heihe-Tengchong Line, represents a significant divide in China’s population distribution. The southeast part of the line is a population gathering area, the transmission of COVID-19 is strong in this area because of strong population mobility and high migration probability. Second, taking Wuhan as the high value center, there is a circular distribution pattern from high to low. Wuhan is not only the city where COVID-19 cases were found for the first time, but also the capital city of Hubei Province. It plays the role of connecting the north and the south, connecting the east and the west in the whole country, and has close ties with the economy, trade, transportation and personnel of the surrounding cities. The scale and location of Wuhan aggravate the panic and uneasiness of the residents in the surrounding cities. Therefore, the ERP presents a circle distribution pattern with Wuhan as the high-value center.

In the decelerating diffusion stage, the ERP of each city has improved, but the distribution characteristics are more obvious. From Figure 7c, one can see that the ERP of Chinese cities has improved, and there are four high-value regions. Among these, the high value areas of ERP are mainly located in Hubei area with Wuhan as the center, Pearl River Delta urban area with Guangzhou-Shenzhen as the center, Yangtze River Delta urban area with Shanghai as the center, and Mudanjiang-Jiamusi area. In the stage of prevention and control stage, the ERP decreased as a whole, but the ERP at border ports and big cities increased. From Figure 7d, one can see that the high value area shows sporadic distribution. The high value areas are mainly distributed in the trade port areas such as Jiamusi, and the big coastal cities such as Shanghai, Guangzhou and Shenzhen. Through commenting text content, the cause of ERP of border port cities and coastal big cities is different. Among them, overseas input is the concerns of the border port urban residents, while the focus along the coastal cities is in large aspects of overseas input, asymptomatic infections and lifestyles.

### 4.2. Direct Influence

Based on the above studies found that ERP has significant space autocorrelation. However, this study focuses on exploring the direct and interaction effects of urban social economic factors on ERP, and discusses the lack of urban development. Therefore, the order logistic regression model is reasonable.

Before performing influencing factors, we first correlate each variable, select the variables related to ERP and incorporate it into the model. Then, we also use the VIF value to verify whether there is multiple common linearities between variables. Finally, we incorporate the variables of COVID-19, economic level, social security and medical facilities into the order logistic model to explore the influencing factors of ERP during COVID-19. As can be seen from Table 2, the fitting effects of the model are all good, and the VIF value of each variable are less than 10, and there is no coordinated or co-linear in an acceptable range between the variables. The result of this model is as follows:

The influencing factors of ERP are different in different stages of COVID-19, and Hypotheses 1 (H1a), 2 (H2) and 3 (H3) are not established in all stages. However, on the whole, we found that confirmed COVID-19 cases and deaths are the catalysts of ERP, and social security and medical facilities are the guarantees to alleviate ERP.

#### 4.2.1. The Stage of Early Dissemination

The cities with a large number of COVID-19 diagnosis and good economic level have higher ERP. The number of people diagnosed with COVID-19 will increase residents’ ERP. As can be seen from Table 2, if the cumulative number of diagnosed people increases by one person, the ERP increased by 0.048 units. At this stage, most cities pay less attention to COVID-19, and the increase in the number of diagnosed patients will aggravate the panic and unease of residents and generate ERP. The average wage of residents will increase their ERP. If the average wage of residents increases by one unit, the ERP increased by 39.855 units. This result is different from that of other stages, mainly because most of the cities with higher average wages are Beijing, Shanghai, Guangzhou, Shenzhen, Hangzhou and so on. Due to high population density and strong mobility, these cities have become the primary spread area of COVID-19. Even if the average wage of the city’s residents is high, it still can’t dispel the panic and insecurity brought about by COVID-19.

Cities with better social security and medical facilities have lower ERPs among their residents. The number of hospitals and unemployment registration in a city can effectively alleviate the ERP. When the number of hospitals and unemployment registration people in cities increased by one unit, the ERPs decreased by 11.121 and 9.765, respectively. It should be emphasized that the unemployment registration in cities in this paper is not the unemployment registration during the COVID-19 period, but is based on the data obtained from the China Urban Statistical Yearbook before the outbreak of COVID-19. This not only reflects the level of urban management and governance, but also exposes the deficiency of urban development, and provides new policy suggestions for resilient urban construction.

#### 4.2.2. The Stage of Accelerating Diffusion

Confirmed COVID-19 cases and deaths significantly increase the ERP. As can be seen from Table 2, if there is a cumulative increase in confirmed COVID-19 cases and deaths, the ERP increased by 0.002 and 0.045 units, respectively. During the Spring Festival travel period, the mobility of the population is greater and the rapid spread of COVID-19 is greater, but compared to confirmed cases, deaths bring more fear and unease to the residents.

Economic level can’t improve the ERP of residents in time for the accelerated spread of COVID-19. As can be seen from Table 2, the impact of the average savings and average wages of residents at this stage is not significant, which shows that in the face of a PHEIC such as COVID-19, the economic level of savings and wages can’t immediately eliminate the panic and unease of residents.

Cities with high population density, endowment insurance and unemployment insurance have a higher ERP of their residents. As can be seen from Table 2, if the population density of the city increases by one unit, the ERP increased by 1.211 units. The main route of transmission of COVID-19 is respiratory droplets and close contact, so it is necessary to maintain social distance to control the spread of the epidemic. Cities with high population density make it more difficult to implement measures such as social isolation, and as a result the sense of panic and fear in the city will gradually increase. The number of residents with endowment insurance and unemployment registration will increase the ERP. If the number of residents with endowment insurance and unemployment registration in a city increases by one unit, the ERP increased by 1.75 and 1.265 units, respectively. The elderly are more likely to suffer from some diseases, and their physical function and resistance are weaker than those of the young. The unemployed lack financial resources, and their daily life is in an unstable state. The elderly and the unemployed are vulnerable groups in need of social assistance, and their ability to resist risks is insufficient. Therefore, their ERP is higher during the spread of COVID-19.

In cities with perfect medical insurance systems, the ERP of residents is lower. If the number of residents with medical insurance in a city increases by one unit, the ERP decreased by 1.976 units. Medical insurance is a social insurance system established to compensate workers for economic losses caused by disease risks. On the one hand, the number of people participating in medical insurance can reflect the perfection of the social security system, on the other hand, it is also the embodiment of the stability of residents’ employment, which provides a buffer for the ERP. If the number of hospitals and doctors in a city is increased by one unit, the ERP decreased by 1.198 and 2.102 units, respectively. The number of hospitals and doctors is the performance of urban medical facilities, and the conditions of medical facilities will reduce residents’ ERP.

#### 4.2.3. The Stage of Decelerating Diffusion

The deaths caused by COVID-19 can significantly increase the ERP. As can be seen from Table 2, if the mortality rate of COVID-19 increases by one unit, the ERP of the city increased by 37.089 units. With the slow growth of confirmed cases and an increase in cured cases, China has begun to promote the resumption of work and the normalization of life in an orderly manner, and the impact of diagnosis on ERP gradually decreased. However, the death rate of each city during COVID-19 makes the residents feel the threat of death intuitively, which further aggravates the panic and unease.

The economic level and medical facilities in the city can effectively reduce the ERP. If the average wage of residents increases by one unit, the ERP of the city decreased by 2.48 units. Economic level is the guarantee for the ordering of production and life, and the average wage of residents is an accelerator of emotional stability. If the number of beds and doctors increases by one unit, the ERP decreased by 1.905 and 1.281 units, respectively. Medical facilities are the city’s shield against COVID-19, so the number of beds and doctors effectively reduces ERP.

#### 4.2.4. The Stage of Prevention and Control

Confirmed cases of COVID-19 will increase ERP. As can be seen from Table 2, if there is an increase in the number of confirmed cases, the ERP increased by 0.006 units. At this stage, cases originating locally has been largely contained, but the risk of an overseas import due to the return of COVID-19 infected persons from abroad has increased dramatically. There is a gradual increase in the number of imported cases from abroad in some large cities and border cities, which will increase ERP of the residents.

Economic level, social security and medical facilities significantly affect ERP. As can be seen from Table 2, if the average wage of residents increases by one unit, the ERP decreased by 2.125 units, which further verifies the correctness of the results of the previous stage. The number of hospitals will aggravate residents’ ERP. If the number of hospitals in the city increases by one unit, the ERP increased by 1.275 units. This is different from the results found for other stages, and the reason may be that the number of hospitals can reflect the economic level and urbanization rate of the city. Since there is an international airport in a big city, it is easier to become a transit station for people who have been infected with COVID-19 to return to China from abroad, and the ERP of residents living in the city will gradually increase. In addition, we can also see from Figure 7d that a high value of ERP is also found in Shanghai, Guangzhou, Shenzhen and other big cities. Endowment insurance and unemployment insurance will aggravate the ERP. As can be seen from the results of Table 2, if endowment insurance and unemployment insurance are increased by one unit, the ERP increased by 1.04 and 1.353 units, respectively.

### 4.3. Interaction Influence

Table 2 shows the direct impact of variables such as COVID-19 and socioeconomic attributes on ERP. In fact, COVID-19 and social variables jointly affect residents’ ERP. So, we then explore the interaction between COVID-19 and socioeconomic factors on ERP. The results of the model are shown in Table 3.

On the whole, the diffusion strength of COVID-19 determines whether it interacts with socioeconomic variables and the degree of interaction. As can be seen from Table 3, in the stage of early dissemination, residents have not yet paid much attention to COVID-19, and COVID-19 does not interact with the socioeconomic variables. In the stage of accelerating diffusion, the interaction between COVID-19 and social security and medical facilities is evident. The interaction between growth rate of confirmed cases and population density, hospital beds, doctors and medical insurance is significant, and their effects were 9.48, 40.9, −25.79 and −12.899, respectively. In the stage of decelerating diffusion, their effects were found to be significant but reduced after the cumulative interaction of average wage, medical insurance and cumulative confirmed cases were taken into account. However, after the interaction between the average wage, doctors and death rate were taken into account, their effects were significant and enhanced at −139.65 and −330.6. This empirical result shows that Hypothesis 3 (H3) is generally valid.

The interaction between cumulative confirmed COVID-19 cases and socioeconomic variables was more obvious than that for deaths. As can be seen from Table 3, in the stage of accelerating diffusion, there were four variables (population density, hospital beds, doctors and medical insurance) that interacted with cumulative confirmed COVID-19 cases, but only one variable (hospital beds) interacted with the number of deaths. In the stage of decelerating diffusion, there was no interaction between socioeconomic variables and deaths. In the prevention and control stage, the socioeconomic variables that interact with COVID-19 variables were evenly distributed in quantity. The number of confirmed cases of COVID-19 was large, the spread was wide and the interaction with socioeconomic variables was greater. This empirical result shows that Hypothesis 3 (H3a) is valid.

The interaction between COVID-19 and social security and medical facilities is more significant. As can be seen from Table 3, in the stage of accelerating diffusion and prevention and control, COVID-19 diagnosis and death status interact with social security and medical facilities (e.g., population density, number of hospitals, number of beds, number of doctors, medical insurance). That is to say, when COVID-19 spreads, the social security and medical facilities are more likely to stabilize ERP. This empirical result shows that Hypothesis 3(H3b) is valid.

## 5. Discussion

The outbreak of COVID-19 has prompted researchers to begin to rebuild urban spaces and to think about the origin of urban planning. In essence, urban planning originates from the response of human beings to health demands, and its purpose is to ensure the safety and health of the city. For example, the epidemic of the Black Death led European countries to strengthen the construction of basic health facilities. The cholera epidemic forced the British government to implement a number of control standards and to provide public services. The Housing and Urban Planning Act promulgated by the British government in 1909 has become one of the symbols of the formal establishment of the modern urban planning system. From historical experience, we find that urban planning is an important way for local governments to ensure urban public health and reduce infectious diseases. In addition, learning from the past is also a source of ideas for improving the quality of a city. Therefore, in the post-pandemic era, the question at hand is how urban planners can create more healthy and resilient cities, particularly in modern cities where the population is highly concentrated, the transportation system is networked, population mobility is high and social communication is diversified.

We found that most of the prior studies on ERP have focused on demographic characteristics. Even though these studies can effectively determine the process by which one is influenced by the other, they are not conducive to the formulation of convenient, efficient and operational planning measures. This study provides empirical evidence for the relationship between ERP and urban socioeconomic factors, and holds that urban economic level, social security and medical resources affect ERP. Our results show that COVID-19 is the catalysts of ERP, and urban socioeconomic variables are the guarantees of ERP. Among these, there are differences in the influence of the urban socioeconomic on ERP. When the spread of COVID-19 accelerates, economic level can’t produce a timely adjustment of ERP, while the regulatory effect of social security and medical resources is more evident. As the socioeconomic factors selected in this paper include urban planning, management and services, our results have scientific validity and provide a new perspective for urban planning.

First, we found that urban medical facilities can effectively alleviate ERP during the COVID-19 epidemic, but they may also increase ERP in this interaction as well. The results of this study are somewhat inconsistent with Hypotheses 3 (H3). There are two possible reasons for this. (1) ERP is affected by many factors, even in cities with sufficient medical resources, the spread of COVID-19 brings great panic and unease to residents. (2) Even though the number of medical facilities is sufficient, the structural layout of medical facilities maybe unreasonable. In the face of health emergencies, residents have little recognition of the surrounding medical resources and think that they can’t resist the crisis. This shows that if we build healthy and resilient cities, we need to start from two aspects: planning and governance. Therefore, we need to consider the following issues when planning: how do we carry out the hierarchical allocation of medical facilities on the premise of adequate medical resources? Should we carry out emergency site facilities planning, and if so, how do we ensure the convenience and utility of the surrounding infrastructure and traffic conditions?

Second, we found that population density increases the sense of panic and insecurity when COVID-19 spreads. Previous studies have verified that there is no direct relationship between high-density cities and infectious disease outbreaks, but high density is nonetheless conducive to the spread of infectious diseases. Cities with high population density have a greater resistance to the implementation of social isolation measures, and residents living in the city will feel panic when faced with an epidemic. In fact, this is not only the impact of high population density, but also the impact of economic, trade and social agglomeration reflected by the agglomeration of population. In the past, cities mostly achieved compact development through high density, mixed land use and convenient transportation, but this study found that population gathering aggravated the panic and unease of residents in the face of PHEIC such as COVID-19. Therefore, we need to think about the following questions in urban planning. How do we manage high-density cities healthily and rationally? How do we find a balance between compact cities and healthy cities?

Most importantly, we found that social security and medical facilities can regulate ERP more effectively than economic conditions can during the COVID-19 epidemic. Social security and medical facilities are the social indicators of the city, which reflect the planning and layout, social governance and management services of the city, and are closely related to the daily life of residents. Therefore, in the post-pandemic era, we not only need to optimize the layout of social facilities, but also need to strengthen the construction of resilient cities. In particular, we need to pay attention to the fine governance of space and recognize the importance of the community in planning and governance- for example, by planning the 15-min community life circle and building a soft community. We must pay attention to the quality of urban planning, education, health care and other livelihood facilities, improve transportation and business-supporting facilities, and truly achieve a healthy and people-oriented urban environment.

## 6. Conclusions

**Figure 8:**
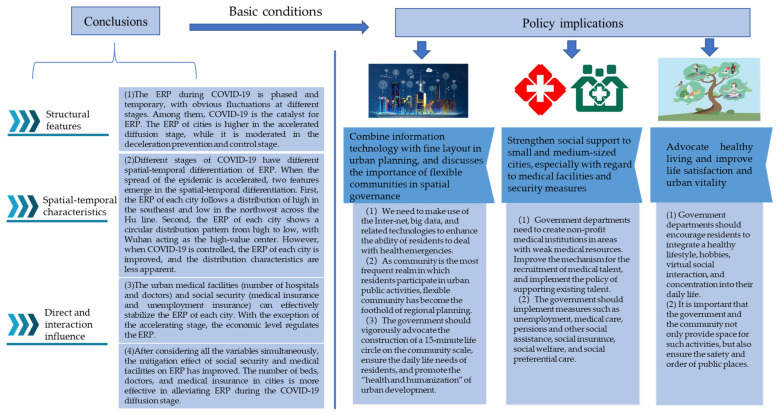
Research conclusions and policy recommendations.

Based on the Weibo comment text, we measured the ERP of each city during the COVID-19 epidemic. After analyzing the structural and spatial-temporal characteristics of ERP, we explored the factors influencing ERP by including COVID-19, economic level, social security and medical facilities into the ordered logistics model. Our findings suggest the following policy recommendations for urban construction. The research conclusions and policy recommendations are shown in Figure 8 and the following:

### 6.1. Conclusions

(1) The ERP during COVID-19 is phased and temporary, with obvious fluctuations at different stages. Among them, COVID-19 is the catalyst for ERP. The ERP of cities is higher in the accelerated diffusion stage, while it is moderated in the deceleration prevention and control stage.

(2) Different stages of COVID-19 have different spatial-temporal differentiation of ERP. When the spread of the epidemic is accelerated, two features emerge in the spatial-temporal differentiation. First, the ERP of each city follows a distribution of high in the southeast and low in the northwest across the Hu line. Second, the ERP of each city shows a circular distribution pattern from high to low, with Wuhan acting as the high-value center. However, when COVID-19 is controlled, the ERP of each city is improved and the distribution characteristics are less apparent.

(3) The urban medical facilities (number of hospitals and doctors) and social security (medical insurance and unemployment insurance) can effectively stabilize the ERP of each city. With the exception of the accelerating stage, the economic level regulates the ERP.

(4) After considering all the variables simultaneously, the mitigation effect of social security and medical facilities on ERP has improved. The number of beds, doctors and medical insurance in cities is more effective in alleviating ERP during the COVID-19 diffusion stage.

### 6.2. Policy Implications

Our findings suggest the following policy recommendations for urban construction:

1. Combine information technology with fine layout in urban planning, and discusses the importance of flexible communities in spatial governance.

In the planning of resilient cities and healthy cities, we need to make use of the Internet, big data and related technologies to enhance the ability of residents to deal with health emergencies. As community is the most frequent realm in which residents participate in urban public activities, flexible community has become the foothold of regional planning. The government should vigorously advocate the construction of a 15-min life circle on the community scale, ensure the daily life needs of residents and promote the “health and humanization” of urban development. In addition, in the post-pandemic era, we need to combine community governance with health and epidemic prevention, focusing on the satisfaction of residents and the high-quality development of cities.

2. Strengthen social support to small and medium-sized cities, especially with regard to medical facilities and security measures.

Social security and medical facilities reflect the spatial governance ability of the city, which can alleviate the ERP of residents in the event of a PHEIC. Due to the lack of funds, talent and resources in small and medium-sized cities, the layout of social facilities within the city is not perfect, and the efficacy of social governance needs to be further strengthened. Therefore, government departments need to create non-profit medical institutions in areas with weak medical resources, improve the mechanism for the recruitment of medical talent and implement the policy of supporting existing talent. In addition, the government should implement measures such as unemployment, medical care, pensions and other social assistance, social insurance, social welfare and social preferential care.

3. Advocate healthy living and improve life satisfaction and urban vitality.

In order to alleviate the ERP of residents in the face of a PHEIC, government departments should encourage residents to integrate a healthy lifestyle, hobbies, virtual social interaction and concentration into their daily life, so as to improve life satisfaction and comfort. It is important that the government and the community not only provide space for such activities, but also ensure the safety and order of public places. These measures can not only effectively adjust the ERP of urban residents, but also improve life satisfaction and urban vitality.

However, this study does have some limitations. First, the ERP of each city was determined by averaging individual data in the city. This can combine the ERP at the individual level with the socioeconomic variables at the urban level, and then provide a corresponding starting point for the construction of resilient cities. However, it relegates the ERP of the individual to less prominence in the analysis, future studies could focus more on the ERP at the individual level. Second, the data source for ERP may be biased because it was taken from a single media platform. Due to the large number of active users and wide range of user groups, Weibo has become an important platform for the acquisition of information and the dissemination of public opinion. The comment text is an important embodiment of Weibo user participation, indicating users’ attitudes and views on public events, and so Weibo comments can reflect their psychological and ERP [45]. In the follow-up study, we plan to make a comprehensive study of the comment data from multiple media platforms.

## Figures and Tables

**Table 1:** Qualitative description of each index.

Types		Variable	Symbol	Indicator Description
COVID-19	Confirmed	Cumulative confirmed cases	CCC	Cumulative number of confirmed cases at a particular stage of the epidemic
	Cases	Growth rate of confirmed cases	GRC	The average growth rate of confirmed cases at a particular stage of the epidemic
	Death	Cumulative death cases	CDC	Cumulative number of deaths at a particular stage of the epidemic
	Cases	Death rate	DR	The average mortality rate at a particular stage of the epidemic
Urban	Economic	Resident deposit balance	RDB	The savings of a city resident over one year
Socio-	Level	Average wage	AW	The average wage of a city resident over a year
economic	Social	Population density	PD	The ratio of the population of a city to the built area
	Security	Endowment insurance	EI	The number of participants with endowment insurance in a city
		Medical insurance	MI	The number of participants with medical insurance in a city
		Unemployment insurance	UI	The number of participants with unemployment insurance in a city
		Unemployment registration	UR	The number of registered unemployed in a city
	Medical	Hospitals	H	The number of hospitals in a city
	Facilities	Hospital beds	HB	The number of hospital beds in a city
		Doctors	D	The number of doctors in a city

**Table 2:** Influencing factors of the environmental risk perception of each city during COVID-19 epidemic.

	Variable	Early Dissemination		Accelerating Diffusion		Decelerating Diffusion		Prevention and Control	
		B	VIF	B	VIF	B	VIF	B	VIF
	1	181.096 ***		14.871 *		−3.582 *		−14.464 *	
	2	174.192 **		11.824 *		−5.626 *		−15.682 *	
	3	171.439 **		8.258 *		−7.546 *		−17.323 *	
COVID-19	CCC	0.048 ***	3.596	0.002 ***	4.287	0.013	2.051	0.006 *	1.703
	GRC	−3.818	3.4	0.882	6.247	−28.425	4.391	15.927	1.879
	CDC	0	2.025	0.045 ***	4.555	−0.077	4.906	0.001	2.058
	DR	0	3.234	10.532	1.721	37.089 **	2.159	−2.387	2.195
Economic	RDB	−0.415	2.014	−0.554	1.925	0.336	1.625	−2.125 **	1.687
Conditions	AW	39.855 **	2.409	1.605	2.95	−2.48 *	2.228	−2.793 *	2.199
Social	PD	−4.174	2.186	1.211 ***	1.603	0.274	1.555	−0.039	1.614
Security	EI	4.526	3.757	1.750 *	4.721	0.199	4.641	1.04 *	4.754
	MI	2.271	1.812	−1.976 ***	6.636	0.535	6.856	−0.198	1.636
	UI	−4.631	4.425	−0.162	0.044	−0.403	2.497	1.353 *	2.411
	UR	−9.765 *	4.452	1.265 **	3.603	0.368	4.415	−0.403	2.848
Medical	H	−11.121 *	2.209	−1.198 *	2.331	0.752	2.324	1.275 **	2.319
Facilities	HB	−0.975	2.596	2.651 **	2.687	−1.905 *	2.88	−0.145	2.772
	D	10.523	3.24	−2.102 **	3.291	1.281 *	3.236	0.152	3.325
MFI	−2LogLikehood	35.432		35.937		703.108		772.966	
	Chi-Square	34.496		29.661		38.366		37.109	
	df	12		11		14		14	

Note: ***, ** and * indicate significant levels at 1%, 5% and 10%, respectively. The meanings of the respective variables are shown in Table 1.

**Table 3:** The interaction of COVID-19 and socioeconomic variables on environmental risk perception.

	Variable	RDB	AW	PD	H	HB	D	EI	MI	UI	UR
early dissemination	CCC										
	CRI										
	CCD										
	MR										
accelerating diffusion	CCC			−0.004 **		−0.017 ***	0.012 *				
	CRI			9.48 ***		40.9 ***	−25.79 **		−12.899 **		
	CCD					0.79 **					
	MR					−447.32 *					
decelerating diffusion	CCC		−0.12 *						0.257 **		
	CRI										
	CCD										
	MR		−139.65 **				−330.6 **				
Prevention And control	CCC										
	CRI			221.7 **	397.3 *						
	CCD				2.52 *				−2.671 *		
	MR			−47.15 **							

Note: ***, ** and * indicate significant levels at 1%, 5% and 10%, respectively. The meanings of the respective variables are shown in Table 1.

## Data Availability

The dataset used in this research are available upon request from the first author. The data are not publicly available due to restrictions, i.e., privacy or ethical.
